# Focal Necrosis and Disturbed Myelination in the White Matter of Newborn Infants: A Tale of Too Much or Too Little Oxygen

**DOI:** 10.3389/fped.2014.00143

**Published:** 2015-01-12

**Authors:** Sven Wellmann, Christoph Bührer, Thomas Schmitz

**Affiliations:** ^1^Division of Neonatology, University Children’s Hospital, Basel, Switzerland; ^2^Department of Neonatology, Charité University Medical Center, Berlin, Germany

**Keywords:** periventricular leukomalacia, white matter disease, hypoxia-inducible factor, oligodendrocyte precursors, myelination

## Abstract

White matter disease in preterm infants comes along with focal destructions or with diffuse myelination disturbance. Recent experimental work with transgenic mice paves the way for a unifying molecular model for both types of brain injury, placing oxygen sensing by oligodendrocyte precursor cells (OPCs) at the center stage. Mice genetically altered to mimic high local oxygen tension in oligodendroglia lineage cells (via deletion of hypoxia-inducible factor, HIF) develop white matter disease resembling cystic periventricular leukomalacia within the first 7 days of life. Mice in which local hypoxia is mimicked in oligodendroglial cells (via genetic inhibition of HIF decay) display arrested OPC maturation and subsequent hypomyelination, reminiscent of the diffuse white matter disease observed in preterm infants and infants with congenital heart disease. These recent experimental findings on oxygen sensing and myelination are awaiting integration into a clinical framework. Gene regulation in response to hyperoxia or hypoxia, rather than oxidative stress, may be an important mechanism underlying neonatal white matter disease.

## Cystic and Diffuse White Matter Disease in Newborn Infants

Brain injury in preterm newborn infants involves both destructive and developmental disturbances (1). Cystic periventricular leukomalacia (cPVL) and diffuse white matter disease (dWMD) are increasingly being viewed as distinct nosological entities rather representing various degrees of severity of the same disorder (2, 3). cPVL is characterized by non-hemorrhagic necrotic lesions leading to non-selective destruction of all cellular elements, including glia and blood vessels (1, 4). These lesions are usually detected as clustered or sporadic porencephalic cysts by cranial ultrasound during the second or third week of life (5). While the incidence of cPVL declined in recent decades, with cPVL affecting now less than 5% of very low birth weight infants (6–9), it remains the strongest predictor of severe cerebral palsy in preterm infants (10). Risk factors for cPVL differ from those of most other prematurity related diseases, as rates of cPVL do not increase with low Apgar scores, lack of antenatal steroids, institution or duration of mechanical ventilation, male sex, or extremely low gestational age (11). Among very low birth weight infants, rates of cPVL are not related to gestational age (6, 11) while the overall risk of neurodevelopmental impairment rises steadily with decreasing gestational age.

Evidence of dWMD has been revealed by advanced magnetic resonance imaging techniques, notably diffusion tensor imaging and magnetic resonance spectroscopy imaging, in many very low birth weight preterm infants (9, 12–14). Moreover, magnetic resonance imaging studies in infants with congenital heart disease have revealed dWMD in a substantial proportion of these infants after neonatal cardiac surgery (15, 16). Some of these lesions, however, are present already before cardiac surgery (17–19). The clinical sequelae of dWMD appear to be more subtle, as compared to those of cPVL, and include cognitive and behavioral problems such as attention deficits, hyperactivity, language impairments, and poor processing of visual information (20–24). Autopsy findings point to disturbed myelination, arising from failure of oligodendrocyte precursor cells (OPCs) to generate myelinating oligodendrocytes, as a key finding in dWMD (8). In contrast to cPVL, there is little axonal degeneration (25) or loss of gray matter neurons in dWMD (26). However, the complexity of dendritic arbors is reduced in dWMD, and the transient failure to establish neuronal connections is thought to underlie persistently altered brain circuitry (2).

## Oxygen-Regulated Gene Expression

Changes of ambient oxygen tension cause a cellular response orchestrated by hypoxia-inducible factors (HIFs) in almost all mammalian cell types (27). The lack of oxygen (hypoxia) strongly activates the master regulators HIF1 and HIF2, normal oxygen tension or an excess in oxygen (hyperoxia) blocks the action of both transcription factors. In detail, HIF1 and HIF2 are two heterodimeric transcription factors consisting each of an oxygen-regulated α-subunit (HIF1α and HIF2α) and an oxygen-insensitive β-subunit. In the presence of oxygen, HIF1α and HIF2α are permanently deactivated by two oxygen-dependent enzymes, asparaginyl and prolyl hydroxylases. Asparagine hydroxylation of a conserved residue inhibits the binding of HIF1α or HIF2α to its co-activator p300/CREB-binding protein (28). Proline hydroxylation targets HIF1α or HIF2α for ubiquitination mediated by the von Hippel–Lindau (VHL) ubiquitin ligase (29, 30), which in turn is followed by proteasomal degradation (31). The enzymatic activity of both, the asparginyl and prolyl hydroxylase, is lowered or even turned off at low oxygen tension, as they both use oxygen as a substrate. Subsequently, spared HIF1α and HIF2α moieties are then able to join oxygen-insensitive β subunits, translocate to the nucleus, and activate a distinct gene response.

## White Matter Disease in Mice with Genetically Altered HIF Metabolism

As intrauterine oxygen tension is difficult to manipulate in experimental animals, researchers at the University of California in San Francisco used mice with either targeted deletions of HIF1α and HIF2α, or lack of VHL-mediated HIF1α/HIF2α decay in the oligodendroglial lineage (32). Embryonic mice unable to express HIF1α and HIF2α in oligodendroglial lineage cells, mimicking high local oxygen tension, had poor vascularization in the white matter before birth, followed by severe neuroaxonal damage and apoptotic cell death of microglia, astroglia, and oligodendroglia by day 4 of life, and ultimately acellular white matter cysts by day 7 of life. In contrast, conditional oligodendroglial HIF1α/HIF2α overexpression mediated by VHL knockout resulted in OPC maturation arrest and subsequent hypomyelination. By using genetically engineered mice with manipulated HIF pathways but kept in normal air, the study specifically investigated the effects of oxygen-sensitive gene regulation, as there was neither oxidative stress associated with excessive oxygen, nor low intracellular energy stores occurring when oxygen supply fails to meet demands. Nevertheless, the delayed OPC differentiation and reduced expression of myelin basic protein observed with VHL knockout-mediated HIF1α/HIF2α overexpression was also observed in wild-type mice exposed to hypoxia (FiO_2_ 0.1) between day 3 and 11 of life.

The investigators went on to show that the regulation of white matter vascularization and oligodendroglial maturation in response to altered HIF1α/HIF2α is mediated by secreted Wnt7a/7b glycoproteins in a paracrine or autocrine fashion, respectively. Wnt7a/7b was previously implicated in oligodendroglial maturation and myelination (33, 34), as well as in white matter vascularization (35), and the new work now demonstrates its regulation by HIF1α/HIF2α. Chronic hypoxia (FiO_2_ 0.1 for 6–72 h) has recently been shown to induce canonical (β-catenin-dependent) Wnt signaling alongside increased cell proliferation in the hippocampus of adult mice (36). In the white matter, the role of Wnt7a/7b is reminiscent of vascular endothelial growth factor (VEGF) as the classical HIF1α/HIF2α target that is involved in angiogenesis and tissue architecture in the gray matter (37).

## Effects of Hypoxia or Hyperoxia on the Brain of Newborn Rodents

In the report that first used the term “periventricular leukomalacia” (38), the authors concluded from the location of the lesions at arterial border zones that either a lack of oxygen or an excess of oxygen might contribute to the pathogenesis of the neonatal white matter disease described (39). Mouse or rat pups subjected to antenatal hypoxia (FiO_2_ 0.1) show increased white matter vessel densities but myelination deficits are only found in rat pups (40). The oligodendroglial maldevelopment and the delay of myelination observed in neonatal rats are aggravated by postnatal hyperoxia (FiO_2_ 0.6 for 7 days) but prevented by slowly increasing FiO_2_ from 0.15 to 0.21 during the first 7 days of life (41). Mice pups reared under low oxygen (FiO_2_ 0.1) between day 3 and day 11 of life display reduced cortical gray and white matter volumes and delayed maturation of OPCs (42). Experimental hypoxia, however, does not elicit the cPVL-type focal necrotic lesions involving all cellular elements, unless cerebral blood supply is drastically perturbed (3). In contrast, the clinical course of preterm infants subsequently diagnosed with cPVL is often inconspicuous, and their mean arterial blood pressures during the first week of life are higher than those of controls (11). A role for cerebral vessel constriction in precipitating cPVL has been inferred from the relationship between cPVL and hypocapnia during the first days of life (11, 43, 44) and the increased rates of cerebral palsy and impaired neurodevelopment associated with neonatal hypocapnia and hyperoxia (45, 46). The work by Yuen et al. (32) suggests that degeneration of white matter vessels mediated by loss of trophic Wnt7a/7b stimulation in response to relative hyperoxia may promote the focal ischemic infarctions that underlie cPVL. cPVL thus joins retinopathy of prematurity (ROP) as a disease of developing arterial blood vessel that is driven by the rapid surge of oxygen tension that invariably follows lung aeration after birth.

The transition from placental to pulmonary oxygenation causes a rapid, drastic and persistent increase of oxygen tension. Oxygen tensions measured in fetal brain are only 10–20% of those found in adult brain (0.08–7.6 mmHg vs. 11–53 mmHg) (47), and the huge oxygen leap after birth necessarily diminishes expression of HIF1α/HIF2α-regulated genes. Human embryonic stem cells can give rise to oligodendroglial lineage cells *in vitro* when cultured at low oxygen of FiO_2_ 0.03 but not at FiO_2_ 0.21 (48, 49). In preterm infants, developmental processes, which physiologically ought to continue for several months under low oxygen conditions (and therefore in the presence of high concentrations of VEGF, Wnt7a/7b, and a multitude of other HIF1α/HIF2α-regulated gene products) are suddenly disturbed or disrupted by premature birth into room air, even if additional supply of oxygen is being avoided. Restraint in using high FiO_2_ with careful monitoring of arterial oxygen saturation by pulse oximetry might have contributed to the decline of cPVL observed over the last decades (6–9).

While experimental hyperoxia in rodents is apt to reproduce several findings of human ROP, exposure to neonatal rats or mice to high oxygen does not necessarily generate the cystic white matter lesions observed with gene-mediated HIF1α/HIF2α-suppression in OPCs acting already before birth. While the brain maturation of newborn rats and mice recapitulates many features of the human situation during the last trimester of pregnancy and therefore that of preterm infants born 2–3 months early, the newborn rodents used in experiments are physiologically adjusted to the perinatal oxygen surge and thus potentially less vulnerable than human preterm babies. Experimental hyperoxia at birth (FiO_2_ 0.6), postnatal day 3 or day 6 (FiO_2_ 0.6), but not day 10, has nevertheless a deleterious effect on the developing white matter in normal rat pups (41, 50). Grown-up mice (30 or 60 days old) previously exposed to hyperoxia (FiO_2_ 0.8) at 6 days of life for 48 h continue to show signs of dWMD when examined by diffusion tensor imaging (51) that reflect myelination abnormalities and consecutive axonopathies (52). Cultured OPCs but not mature oligodendrocytes undergo apoptotic cell death when directly subjected to high oxygen (50, 53) but it is unknown whether this also involves HIF1α/HIF2α-mediated downregulation of trophic factors or other non-HIF1α/HIF2α-mediated mechanisms.

## Concluding Remarks

In sum, the immature white matter of both human beings and rodents is highly sensitive to altered oxygen tension during a critical developmental window prior to the onset of myelination. Both too much and too little oxygen can cause damage by profoundly altering expression of various genes, unrelated to any oxidative stress. These two conditions may occur sequentially in the same brain, as reduced vessel density after high oxygen subsequently leads to low oxygen supply at a time when demand is on the rise. Meticulous attempts to avoid extremes of oxygen tension in the brains of preterm infants and in infants with congenital heart disease undergoing surgery, possibly aided by directly monitoring cerebral oxygenation (54), will hopefully further preserve their white matter integrity.

## Conflict of Interest Statement

The authors declare that the research was conducted in the absence of any commercial or financial relationships that could be construed as a potential conflict of interest.
